# A retrospective study of microscopic polyangiitis patients presenting with pulmonary fibrosis in China

**DOI:** 10.1186/1471-2466-14-8

**Published:** 2014-01-28

**Authors:** Hui Huang, Yan xun Wang, Chun guo Jiang, Jia Liu, Ji Li, Kai Xu, Zuo jun Xu

**Affiliations:** 1 Department of Respiratory Medicine, Peking Union Medical College Hospital, Chinese Academy of Medical Sciences & Peking Union Medical College, #1 Shuaifuyuan Street, Beijing, Dongcheng District 100730, China; 2 Pathological Department, Peking Union Medical College Hospital, Chinese Academy of Medical Sciences & Peking Union Medical College, #1 Shuaifuyuan Street, Beijing, Dongcheng District 100730, China; 3 Radiological Department, Peking Union Medical College Hospital, Chinese Academy of Medical Sciences & Peking Union Medical College, #1 Shuaifuyuan Street, Beijing, Dongcheng District 100730, China

**Keywords:** Pulmonary fibrosis, Systemic vasculitis, ANCA

## Abstract

**Background:**

Pulmonary involvement is a common feature of MPA. Although alveolar hemorrhage is the most common pulmonary manifestation of MPA, a few recent studies have described instances of MPA patients with pulmonary fibrosis. Pulmonary fibrosis was seen to predate, be concomitant with, or occur after the diagnosis of MPA. The goal of this study was to describe the clinical features and prognosis of microscopic polyangiitis (MPA) patients whose initial respiratory presentation was pulmonary fibrosis.

**Methods:**

We conducted a retrospective analysis of 19 MPA patients who presented with pulmonary fibrosis at Peking Union Medical College Hospital between 1990 and 2012.

**Results:**

Of 67 total MPA cases, 19 patients presented with pulmonary fibrosis. There were 8 males and 11 females, with a median age of 63.6 years. Common clinical manifestations included fever (89.5%), cough (84.2%), dyspnea (78.9%) and velcro rales (84.2%). Eleven patients experienced weight loss, several had kidney involvement, and most had an increased erythrocyte sedimentation rate and C-reactive protein. All were positive for myeloperoxidase-anti-neutrophil cytoplasmic antibody (ANCA), with 6 patients being positive at the time of their initial diagnosis of pulmonary fibrosis. Every patient had typical features of usual interstitial pneumonia on High-resolution CT. All were treated with corticosteroids and cyclophosphamide, which lead to an improvement in twelve cases. One of the remaining patients progressed slowly, whereas six died.

**Conclusions:**

Patients with MPA, who also presented with pulmonary fibrosis in our cohort, were more likely to be older, female, and have extrapulmonic involvement. Most patients had a delayed positive ANCA. Corticosteroids plus cyclophosphamide was the remission-induction treatment scheme for all cases. The current prognosis for MPA patients with pulmonary fibrosis appears to be poor, suggesting that they may be candidates for new therapies.

## Background

Microscopic polyangiitis (MPA) is a common cause of pulmonary-renal syndrome [1]. Although the kidney is the most commonly involved organ in MPA, a substantial proportion of patients (25–55%) may also have lung involvement [2]. Although the characteristic clinical manifestation of MPA in the lung is pulmonary alveolar hemorrhage, a few recent small studies and case reports have described instances of MPA patients with pulmonary fibrosis [3-7]. In these studies, pulmonary fibrosis was seen to predate, be concomitant with, or occur after the diagnosis of MPA. In order to facilitate the recognition, diagnosis, and prognosis of this uncommon disease, we now describe the clinical, radiological, and immunological characteristics, as well as the outcomes of 19 MPA patients whose initial lung manifestation was pulmonary fibrosis.

## Methods

We used a computer-assisted search to identify 67 patients hospitalized with MPA (according to the 2012 revised International Chapel Hill Consensus Conference nomenclature of vasculitides [8]) at Peking Union Medical College Hospital between January 1st 1990 and January 1st 2012. A review of the medical records and radiological images revealed 19 cases whose initial respiratory manifestation was consistent with a usual interstitial pneumonia (UIP) pattern or a possible UIP pattern according to the ATS/ERS/JRS/ALAT statement [9]. Informed consent was obtained from every subject and/or their family. This study was approved by the Peking Union Medical College Hospital review board.

Patients’ characteristics were recorded from their medical records, and their high-resolution computed tomography (CT) images were downloaded from our hospital’s image bank. Three radiological specialists conducted a consensus reading of the CT images. Patients’ age, sex, symptoms at presentation, physical examination, laboratory and radiological findings, treatment, and outcomes were analyzed. Data are expressed as mean ± standard deviation (SD) for continuous variables and as percentages for categorical variables.

## Results

### Demographics and clinical manifestations

The clinical characteristics of the 19 MPA patients included in this study cohort are summarized in Table 1. The study group consisted of 8 male and 11 female patients with a mean age of 63.6 years (range between 44–75 years of age). Only 2 patients were younger than 40 years of age, and 3 patients were between 50 and 60 years of age. As HRCT was widely used in our hospital during the winter of 2003, three cases without HRCT, which were admitted before 2004 were excluded. All of the patients in the cohort were admitted between 2004 and 2011.

**Table 1:** Demographics and clinical features of the patients in this study

**Case no**	**Age (yr)/sex**	**Fever**	**Weight loss (kg)**	**Dyspnea**	**Hemoptysis**	**Renal disease**	**Arthralgia**	**Myalgia fatigue**	**Rash**	**ANCA Titer/ MPO**	**Outcome**
1	66/F	+	5	+	-	-	-	Myalgia	-	1:40/69	Progressed
2	71/M	+	2	+	-	Hematuria CRF	+	Myalgia	-	1:80/131	Improved
3	68/M	+	0	+	-	Hematuria, Proteinuria	-	-	-	1:80/177	Improved
4	44/M	+	0	+	-	Ematuria	+	Myalgia	-	1:640/322	Improved
5	62/M	+	12	+	-	Hematuria	-	Fatigue	-	1:80/98	Improved
6	64/F	+	4	+	-	-	+	-	-	1:40/66	Improved
7	64/F	+	0	+	+	-	-	-	Eczema- like rash	1:40/56	Improved
8	74/F	+	6	-	-	Hematuria	-	Myalgia	-	1:160/195	Died
9	47/F	+	0	-	-	-	-	Myalgia fatigue	-	1:40/48	Improved
10	75/F	+	0	-	-	RPGN	-	-	-	1:80/116	Improved
11	51/M	+	0	+	-	ARF	-	-	-	1:320/243	Died
12	72/M	+	15	-	+	ARF	-	-	-	1:40/80	Died
13	50/M	-	10	+	+	-	-	-	-	1:160/289	Died
14	59/F	+	0	+	-	-	-	-	-	1:80/127	Improved
15	68/F	+	5	+	+	Proteinuria	-	Myalgia	-	1:80/115	Improved
16	63/F	+	0	+	-	-	-	-	-	1:80/139	Improved
17	70/F	-	3	+	-	Hematuria, proteinuria CRF	-	Myalgia fatigue	Purpura- like rash	1:80/188	Improved
18	69/F	+	3	+	-	Hematuria	+	Myalgia	-	1:160/356	Improved
19	72/M	+	10	+	-	Hematuria, proteinuria	-	-	Purpura- like rash	1:160/312	Died

*M* male, *F* female, *CRF* chronic renal failure, *RPGN* rapidly progressive glomerulonephritis, *ARF* acute renal failure, proteinuria: urine protein > 150 mg/24 h.

Constitutional symptoms included fever (89.5%, with 2 patients having high-grade fever), weight loss (57.9%, mean 6.8 kg, range 2–15 kg), fatigue (47.4%), and loss of appetite (26.3%). There were 7 cases (36.8%) that had fever before the diagnosis of pulmonary fibrosis. Among these 7 cases, one patient’s fever was relieved after antibiotics, while the others were relieved after the administration of corticosteroids. The other 12 cases (63.2%) had fever after the diagnosis of pulmonary fibrosis and had a mean delay of 7.3 months (range of 2 months to 13 years). Antibiotics were not effective for the fever of these patients, but corticosteroids were effective.

Respiratory symptoms included cough (84.2%), sputum (68.4%), hemoptysis (21.1%) and dyspnea (78.9%). Only two patients had clubbing, whereas Velcro rales could be heard in the majority of cases (84.2%). At least one extrapulmonic organ was affected in most cases (Table 1), with nephropathy being the most common. Eye involvement, including iridocyclitis, scleritis, and keratitis, was reported in 3 cases. Two cases had central nervous system disease, with demyelination seen by magnetic resonance imaging (MRI). One patient had a peptic ulcer and presented with gastrointestinal bleeding.

### Laboratory tests

All patients in the cohort were initially given complete blood counts (CBC), urinalysis, biochemical tests (including liver, renal function, and electrolyte levels), erythrocyte sedimentation rate (ESR), C-reactive protein (CRP), anti-nuclear antibody (ANA), anti-extractable nuclear antigen (ENA) and anti-neutrophil cytoplasmic antibody (ANCA) analyses. Peripheral white blood cell counts ranged from 5.6 × 10^9^/L to 31.2 × 10^9^/L (mean 11.3 × 10^9^/L), with neutrophils comprising the largest portion (mean, 76.2%, range, 50.1-95.2%). Eight cases had variable anemia with hemoglobin ranging from 8.2 to 10.8 g/L. Most patients had elevated ESR and CRP (94.7% and 78.9%, respectively), and only 7 cases (36.8%) had normal urinalysis and/or renal function (Table 1). Furthermore, all 19 patients (100%) were myeloperoxidase (MPO)-ANCA-positive (range, 43 – > 200 EU; healthy normal control values are < 10 EU as measured by ELISA), with 6 patients (31.6%) being ANCA-positive at the time of diagnosis of pulmonary fibrosis. In the other 13 cases, patients became ANCA-positive during the follow-up period for pulmonary fibrosis and had a mean delay of 9.5 months (range, 4 months to 13 years). Four patients did not become ANCA-positive until more than 12 months after the initial diagnosis of pulmonary fibrosis. Anti-proteinase-3 and anti-glomerular basement membrane antibodies were negative in all patients.

Bronchoalveolar lavage (BAL) was performed on 5 patients, and BAL fluid analysis showed neutrophil percentages of 4%, 9%, 16%, 32%, and 38% with lymphocyte percentages of 6%, 10%, 15%, 40%, and 41%, respectively. None of these patients had an elevated eosinophil level.

### CT scan images and pulmonary function tests

**Figure 1:**
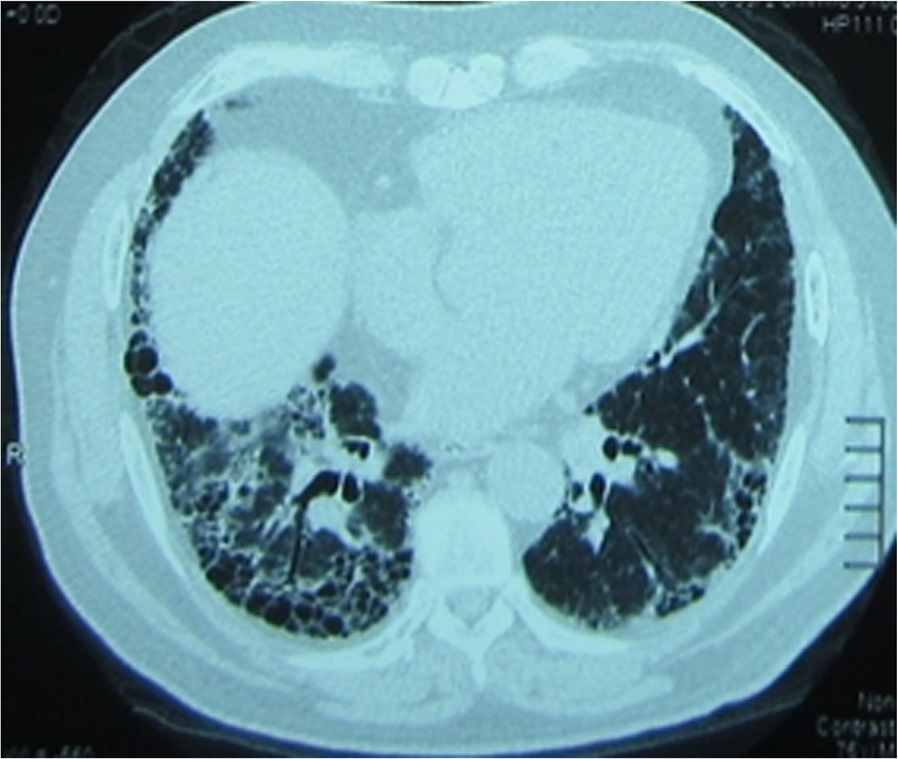
**Patient with pulmonary fibrosis had positive ANCA at her initial screening: A 64-year-old woman complained of cough and exertional dyspnea.** She had UIP-pattern on chest CT: reticular opacities and honeycombing with basal and peripheral distribution. But her ANCA showed a myeloperoxidase of 196 EU and she was diagnosed with MPA. She was then treated with corticosteroids and cyclophosphamide and had a good outcome.

**Figure 2:**
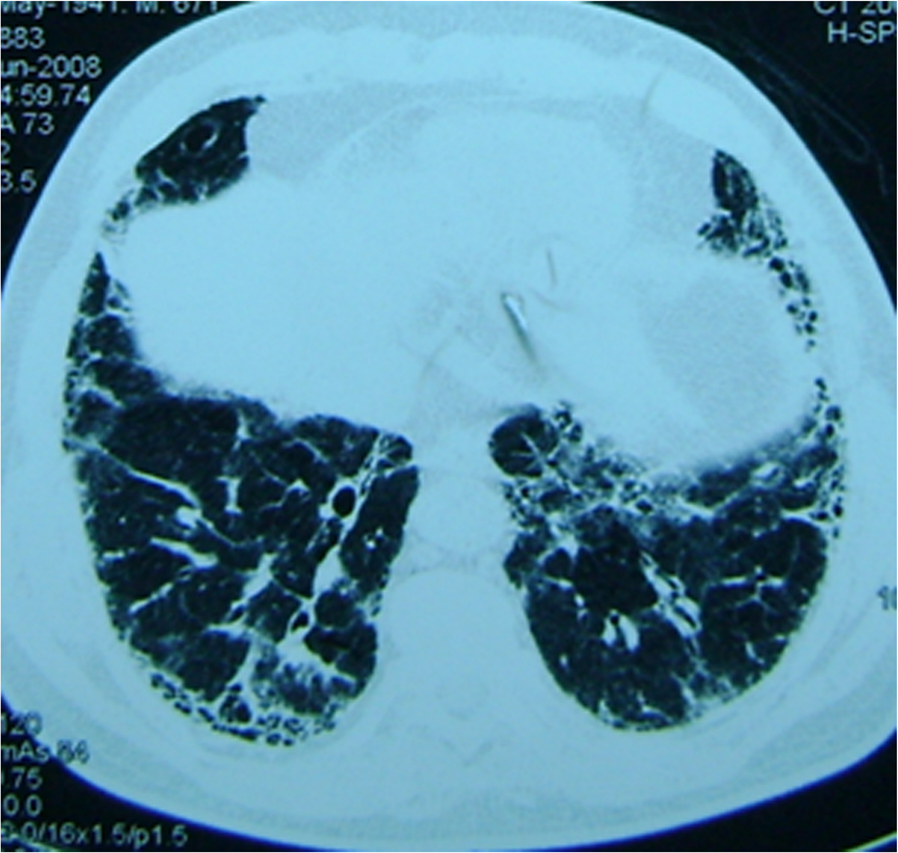
**Patient with pulmonary fibrosis had delayed final diagnosis of MPA: A 71-year-old male diagnosed with idiopathic pulmonary fibrosis by surgical lung biopsy.** A chest CT showed reticular shadows in the basal aspect of each lung. He was treated with 1800 mg/d N-Acetylcysteine. Fourteen months later, his urinalysis revealed hematuria secondary to glomerulonephritis. And he had an intermittent fever for 2 months with an ANCA of 1:40. Percutaneous renal biopsy showed focal segmental necrotizing glomerulonephritis and glomerular crescents. He was diagnosed with MPA and treated with corticosteroids and cyclophosphamide.

Since all 19 patients were diagnosed with MPA after 2004, they all had high-resolution CT images (HRCT; Figure 1 and 2) (Informed written consent was obtained for the publication of individual personal information from these two patients). The most common feature in each patient was reticular shadows, with approximately 40-70% of the whole lung involved. Additionally, some patients (10/19) had honeycomb lesions (approximately 5-31% of the whole lung), and 2 patients had minimal ground-glass opacities (both were less than 5%). All of the shadows were predominant in both lower lobes of the lungs and had outer zone predominance. Eleven patients received pulmonary function testing (PFT) upon the diagnosis of pulmonary fibrosis. Among them, PFT disclosed a restrictive pattern of ventilation and decreased gas diffusion, with a total lung capacity from 48.2% to 72.8% and a carbon monoxide transfer factor (DLCO) from 30% to 76%.

### Echocardiography and electromyography

Echocardiography was carried out in fourteen patients, revealing pulmonary artery hypertension in 4 (mean, 55 mmHg; range 45-75 mmHg; 1 mmHg = kPa). However, all 14 patients had a normal ejection fraction. Electromyography results were available for 7 patients, which revealed peripheral neuropathy in 4 patients.

### Tissue biopsy and pathological results

Four patients received a transbronchial lung biopsy, with fibroblast foci found in only one case. Another patient was diagnosed with usual interstitial pneumonia following a surgical lung biopsy. Kidney biopsies were carried out in 2 cases, and both showed segmental and necrotizing glomerulonephritis. Finally, 4 patients underwent muscle biopsies, revealing vasculitis in 1 patient.

### Pulmonary fibrosis and MPA

Within 2 to 6 weeks of their initial diagnosis, six of the patients diagnosed with MPA had pulmonary fibrosis. Pulmonary fibrosis was seen to predate the diagnosis of MPA for the other 13 cases, with a mean delay of 9.5 months (range, 4 months to 13 years). Two of the cases had been misdiagnosed as idiopathic pulmonary fibrosis for more than 2 years. They were treated with BIBF1120 (a new drug made by Boehringer Ingelheim in clinical trials) for seven months and pirfenidone for 12 months respectively. Antibiotics were prescribed for fever, but were ineffective. After stopping BIBF1120 and pirfenidone, both patients still had a fever. Both patients had microscopic hematuria and kidney biopsies were arranged for both of them. Both showed segmental and necrotizing glomerulonephritis. Within one and four months of the biopsy, they had a positive ANCA respectively. None of the cases had pulmonary fibrosis after the diagnosis of MPA.

### Treatment and outcomes

The median follow-up was 29.9 months (range, 8–93 months). All patients in the cohort were treated with corticosteroids and cyclophosphamide in the remission stage. According to the 2000 guidelines of IPF, all of the patients who had pulmonary fibrosis before the diagnosis of MPA were given small dosages of corticosteroids: 0.5-0.6 mg/kg/d prednisone was prescribed. With the diagnosis of MPA, the initial doses ranged from 1 mg/kg to 2 mg/kg per day for 16 of the cases. Three cases with acute renal failure were given intravenous methylprednisolone 1000 mg for 3 days and tapered to prednisone 1 mg/kg per day. The initial dose of cyclophosphamide was 2 mg/kg/d every day for at least 3 months, and then tapered to 1–1.5 mg/kg per day. Some of the cases that were misdiagnosed with IPF were treated with AZA. Additionally, 3 patients were treated with hemofiltration or hemodialysis due to renal failure. Twelve cases had a lessening of symptoms, with another patient progressing slowly, and six patients dying. Of the six patients that died, two had an exacerbation of their pulmonary fibrosis; both of them had diffuse alveolar hemorrhage that was diagnosed by BAL. One of the two patients also had complicated acute renal failure. The cause of death in the remaining four cases was severe pulmonary infection and respiratory failure, which occurred during the steroid and immunosuppressant treatment period.

## Discussion

Nada et al. first reported ANCA-associated pulmonary fibrosis in 1990 [10]. Since then, several additional reports have described ANCA-, MPO-ANCA-, and MPA-associated pulmonary fibrosis [3-7,11,12]. Although pulmonary fibrosis can be present at the time of MPA diagnosis, or manifest months to years prior to or after MPA diagnosis, cases in our cohort all had pulmonary fibrosis as an initial pulmonary manifestation of MPA. About two thirds of them had a delayed diagnosis of MPA of more than 4 months.

MPA has a slight male predominance (male-to-female ratio: 1.8:1), with an average age of onset between 50–60 years [2]. Our cohort had an older female predominance, which differs from the normal distribution of MPA patients both with and without pulmonary fibrosis; it is also different from most prior studies of MPA-associated pulmonary fibrosis. However, Eschun, Mink, and Sharma’s report describing pulmonary fibrosis as a presenting MPA manifestation reported a 1:1 male-to-female ratio (3 men, 3 women) [3]. Furthermore, in our study, there were no dramatic differences in HRCT manifestations and pulmonary function characteristics between patients with MPA-associated pulmonary fibrosis and typical IPF patients. Additionally, according to the 2011 IPF guidelines, a diagnosis of IPF can be made by HRCT findings suggestive of IPF. Based on the guidelines and our results, we should include MPA in the differential diagnosis of a cause of IPF, especially when the patient is female. This also indicates the need for a follow-up ANCA test.

In our study of 19 cases, clubbing was rarely present, whereas extrapulmonary manifestations were more common. Most patients had constitutional symptoms, such as fever and weight loss similar to previous reports [7]. Elevated CRP and ESR and abnormal urinalysis and/or renal function were also common in our patients. This indicates that pulmonary fibrosis patients who present without clubbing but present with extrapulmonary manifestations and/or renal damage (especially when accompanied by fever), may indicate the need for an ANCA test for the final diagnosis. These patients should not be considered to only have pulmonary fibrosis as a complication of a lung infection.

Pulmonary involvement occurs in 30-70% of MPA cases [11], with the classic pulmonary manifestation being diffuse alveolar hemorrhage caused by pulmonary capillaritis [2,13]. Some studies have suggested that subclinical intraalveolar hemorrhage may play a role in the development of pulmonary fibrosis in MPA [14-16]. However, in our study only 21.1% of patients had hemoptysis, and none of the lung biopsies showed alveolar hemorrhage. This is in agreement with Hervier et al., [5] and indicates that pulmonary hemorrhage might not be the root cause of MPA-associated fibrosis.

In accordance with previous reports [3,5,11], all of our cases were positive for MPO-ANCA. Most cases of other published studies were also MPO-ANCA positive. Diffuse alveolar hemorrhage, which has been reported in 12-55% of MPA patients, has been the classic pulmonary manifestation. Furthermore, 50-75% of cases are ANCA positive [2]. Using an animal model for anti-MPO-associated pulmonary vasculitis, Foucher et al. [17] found that MPO-ANCA plays a pathogenic role for generalized pulmonary tissue injury in ANCA-associated vasculitis. However, whether MPO positivity is a predisposing factor for pulmonary fibrosis in MPA patients is not yet understood.

Similar to the results in the studies by Eschun et al [3] and Foulon et al. [4], most of our cohort cases had pulmonary fibrosis before the diagnosis of MPA. About one third of cases were diagnosed with MPA during the search for secondary causes of pulmonary fibrosis. That was different from the Tzelepis’ [7] and Hervier’s [5] study. In their cohort, pulmonary fibrosis was present in 36- 67% patients at the time of diagnosis, and 21-25% patients had pulmonary fibrosis preceding the other findings of MPA.

An induction of remission treatment followed by maintenance therapy is recommended for most MPA patients, especially if they present with glomerulonephritis. While 90% of MPA patients achieve remission on this regimen, there is no consensus on a therapy regimen for MPA patients whose main manifestation is pulmonary fibrosis. In one study, none of the 12 patients improved while on immunosuppressive therapy [5]. In another study, corticosteroids and/or another immunosuppressive drug were given for most patients with MPO-ANCA-positive pulmonary fibrosis [11]. However, their prognosis was similar to IPF patients. Finally, one study showed significantly greater all-cause mortality among MPA patients with pulmonary fibrosis, leading the authors to postulate that patients with MPA-associated pulmonary fibrosis have an extremely poor prognosis [7]. However, in a study by Arulkumaran et al. [12], there was no difference in mortality between patients with MPA vasulitis alone and MPA-associated interstitial lung disease, leaving the impact of MPA-associated pulmonary fibrosis on mortality uncertain. Although all patients in our study were treated with corticosteroids and immunosuppressant therapy, one patient progressed slowly, two patients had an exacerbation of pulmonary fibrosis, and 6 patients died. Mycophenolate mofetil [18] and rituximab [19] have previously been used as alternatives to cyclophosphamide for MPA patients (most of them in patients with renal manifestations), and the results have shown that they are non-inferior alternatives to cyclophosphamide. These new approaches might be tried in the future to improve the prognosis of MPA-associated pulmonary fibrosis.

Our study had limitations because of the retrospective nature, reliance on clinical notes for data collection, the small patient numbers, and the severity of some cases. First, not all patients had undergone extensive pulmonary screening, including pulmonary function testing (PFT) and bronchoalveolar lavage. This resulted in an underestimation of the character and possibly the prognostic value of the PFT and BAL abnormalities seen in patients with pulmonary fibrosis of MPA. In addition, not all of the patients received a kidney biopsy. Because of this we could not describe the type of renal involvement for them. Although all cases were treated with corticosteroids and cyclophosphamide, the treatment regimes were heterogeneous. Given the rarity of this condition, multicenter collaboration was required to pool the numbers and to clarify the epidemiology, clinical features, optimal treatment strategies, and prognosis for Chinese MPA patients who had pulmonary fibrosis.

## Conclusions

Patients with MPA whose initial respiratory presentation was pulmonary fibrosis in our cohort, were more likely to be older, female, and have extrapulmonic involvement. Most patients had a delayed positive ANCA. The current prognosis for MPA patients with pulmonary fibrosis appears to be poor. New drugs may also be tried in order to improve prognosis of these patients.

## Abbreviations

MPA: Microscopic polyangiitis; UIP: Usual interstitial pneumonia; CT: Computed tomography; MRI: Magnetic resonance imaging; ESR: Erythrocyte sedimentation rate (ESR); CRP: C-reactive protein (CRP); ANA: Anti-nuclear antibody; ENA: Anti-extractable nuclear antigen; ANCA: Anti-neutrophil cytoplasmic antibody; MPO: Myeloperoxidase; BAL: Bronchoalveolar lavage; PFT: Pulmonary function test; DLCO: Carbon monoxide transfer factor.

## Competing interests

The authors declare they have no competing interest.

## Authors’ contributions

XZJ served as the guarantor of the paper, and takes responsibility for the integrity of the work as a whole. HH and WYX conceived the study, and participated in its design and coordination. HH performed the statistical analysis and drafted the manuscript. WYX, JCG, LJ (Liu Jia), LJ (Li Ji) and XK participated in data collection. All authors read and approved the final manuscript.

## Pre-publication history

The pre-publication history for this paper can be accessed here:

http://www.biomedcentral.com/1471-2466/14/8/prepub

## References

[B1] Niles JL Böttinger EP Saurina GR Kelly KJ Pan G Collins AB McCluskey RT The syndrome of lung hemorrhage and nephritis is usually an ANCA-associated condition Arch Intern Med 1996 156 440 445 10.1001/archinte.1996.00440040118013 8607730

[B2] Chung SA Seo P Microscopic polyangiitis Rheum Dis Clin North Am 2010 36 545 558 10.1016/j.rdc.2010.04.003 20688249 PMC2917831

[B3] Eschun GM Mink SN Sharma S Pulmonary interstitial fibrosis as a presenting manifestation in perinuclear antineutrophilic cytoplasmic antibody microscopic polyangiitis Chest 2003 123 297 301 10.1378/chest.123.1.297 12527637

[B4] Foulon G Delaval P Valeyre D Wallaert B Debray MP Brauner M Nicaise P Cadranel J Cottin V Tazi A Aubier M Crestani B ANCA-associated lung fibrosis: analysis of 17 patients Respir Med 2008 102 1392 1398 10.1016/j.rmed.2008.04.023 18640019

[B5] Hervier B Pagnoux C Agard C Haroche J Amoura Z Guillevin L Hamidou MA French Vasculitis Study Group Pulmonary fibrosis associated with ANCA-positive vasculitides. Retrospective study of 12 cases and review of the literature Ann Rheum Dis 2009 68 404 407 10.1136/ard.2008.096131 18957485

[B6] Nozu T Kondo M Suzuki K Tamaoki J Nagai A A comparison of the clinical features of ANCA-positive and ANCA-negative idiopathic pulmonary fibrosis patients Respiration 2009 77 407 415 10.1159/000183754 19077381

[B7] Tzelepis GE Kokosi M Tzioufas A Toya SP Boki KA Zormpala A Moutsopoulos HM Prevalence and outcome of pulmonary fibrosis in microscopic polyangiitis Eur Respir J 2010 36 116 121 10.1183/09031936.00110109 19926741

[B8] Jennette JC Falk RJ Bacon PA Basu N Cid MC Ferrario F Flores-Suarez LF Gross WL Guillevin L Hagen EC Hoffman GS Jayne DR Kallenberg CG Lamprecht P Langford CA Luqmani RA Mahr AD Matteson EL Merkel PA Ozen S Pusey CD Rasmussen N Rees AJ Scott DG Specks U Stone JH Takahashi K Watts RA 2012 Revised international chapel hill consensus conference nomenclature of vasculitides Arthritis Rheum 2013 65 1 11 23045170 10.1002/art.37715

[B9] Raghu G Collard HR Egan JJ Martinez FJ Behr J Brown KK Colby TV Cordier JF Flaherty KR Lasky JA Lynch DA Ryu JH Swigris JJ Wells AU Ancochea J Bouros D Carvalho C Costabel U Ebina M Hansell DM Johkoh T Kim DS King TE Jr Kondoh Y Myers J Müller NL Nicholson AG Richeldi L Selman M Dudden RF Griss BS Protzko SL Schünemann HJ ATS/ERS/JRS/ALAT Committee on Idiopathic Pulmonary Fibrosis An official ATS/ERS/JRS/ALAT statement: idiopathic pulmonary fibrosis: evidence-based guidelines for diagnosis and management Am J Respir Crit Care Med 2011 183 788 824 10.1164/rccm.2009-040GL 21471066 PMC5450933

[B10] Nada AK Torres VE Ryu JH Lie JT Holley KE Pulmonary fibrosis as an unusual clinical manifestation of a pulmonary renal vasculitis in elderly patients Mayo Clin Proc 1990 65 847 856 10.1016/S0025-6196(12)62575-0 2195245

[B11] Homma S Matsushita H Nakata K Pulmonary fibrosis in myeloperoxidase antineutrophil cytoplasmic antibody-associated vasculitides Respirology 2004 9 190 196 10.1111/j.1440-1843.2004.00581.x 15182268

[B12] Arulkumaran N Periselneris N Gaskin G Strickland N Ind PW Pusey CD Salama AD Interstitial lung disease and ANCA-associated vasculitis: a retrospective observational cohort study Rheumatology (Oxford) 2011 50 2035 2043 10.1093/rheumatology/ker236 21873269

[B13] Colby TV Fukuoka J Ewaskow SP Helmers R Leslie KO Pathologic approach to pulmonary hemorrhage Ann Diagn Pathol 2001 5 309 319 10.1053/adpa.2001.27923 11598860

[B14] Schnabel A Reuter M Csernok E Richter C Gross WL Subclinical alveolar bleeding in pulmonary vasculitides: correlation with indices of disease activity Eur Respir J 1999 14 118 124 10.1034/j.1399-3003.1999.14a20.x 10489838

[B15] Al Riyami BM Al Kaabi JK Elagib EM El Khatim HS Woodhouse NJ Subclinical pulmonary haemorrhage causing a restrictive lung defect in three siblings with a unique urticarial vasculitis syndrome Clin Rheumatol 2003 22 309 313 10.1007/s10067-003-0738-x 14579162

[B16] Birnbaum J Danoff S Askin FB Stone JH Microscopic polyangiitis presenting as a “pulmonary-muscle” syndrome: is subclinical alveolar hemorrhage the mechanism of pulmonary fibrosis? Arthritis Rheum 2007 56 2065 2071 10.1002/art.22633 17530647

[B17] Foucher P Heeringa P Petersen AH Huitema MG Brouwer E Tervaert JW Prop J Camus P Weening JJ Kallenberg CG Antimyeloperoxidase-associated lung disease. An experimental model Am J Respir Crit Care Med 1999 160 987 994 10.1164/ajrccm.160.3.9807139 10471629

[B18] Han F Liu G Zhang X Li X He Q He X Li Q Wang S Wang H Chen J Effects of mycophenolate mofetil combined with corticosteroids for induction therapy of microscopic polyangiitis Am J Nephrol 2011 33 185 192 10.1159/000324364 21311184

[B19] Roll P Ostermeier E Haubitz M Lovric S Unger L Holle J Kötter I Henes JC Bergner R Rubbert-Roth A Specker C Schulze-Koops H Müller-Ladner U Fleck M Burmester GR Hiepe F Heitmann S Aringer M Fischer-Betz R Dörner T Tony HP Efficacy and safety of rituximab treatment in patients with antineutrophil cytoplasmic antibody-associated vasculitides: results from a German registry (GRAID) J Rheumatol 2012 39 2153 2156 10.3899/jrheum.120482 22984269

